# A Case of Vulvitis in a Little Girl, Terminating Fatally; with Remarks

**Published:** 1867-10

**Authors:** A. Given

**Affiliations:** Louisville, Ky.


### ARTICLE III.

*A Case of Vulvitis in a Little Girl Terminating Fatally; with Remarks.* By A. Given, M. D., of Louisville, Ky.

Under the head of "Infantile Leucorrhœa," Dr. Churchill describes two grades of inflammation affecting the vulva of children. The first is characterized by local uneasiness, itching, and scalding on making water; the mucous membrane is found inflamed. After a few days, there is observed a leuchorrhœal discharge; the labia swell, inflame, and often become excoriated. The child is feverish and uneasy; the distress increasing with the progress of the disease—the smarting and scalding are severe, and the little patient cannot walk without pain. These symptoms, however, readily subside by local applications. The second grade has prevailed as an epidemic in some parts of Europe, and assumed a malignant type. Mr. Kinder Wood gave a very graphic description of the cases observed by him in 1815, from which Churchill quotes as follows: "The patients were from one to six years of age. Of twelve who were attacked, only two recovered. The inflammation of the labia was preceded by rigors, pain in the head, dullness, nausea, loss of appetite, thirst, &c. The distress of the patient on passing urine first attracted attention; and on examination, the labia were found inflamed, swollen, and of a dark color. Very soon the parts within the vulva became affected, and, from the thin discharge," Mr. Wood thinks "it is probable that the lower portion of the vagina was involved. The process of ulceration set in rapidly, twenty-four hours sufficing for the production of vesication within the labia; and when these burst the denuded surfaces coalesced and formed large ulcers. The discharge then became dark colored, copious, and offensive, irritating the neighboring parts, and favoring the extention of the disease to the thighs, perineum, and anus. After the occurrence of ulceration, the external organs of generation are progressively destroyed, the peculiar pallor of the countenance increases, the pulse becomes quick and weak, the appetite fails, the discharge from the parts increases and becomes more and more offensive, till the patient is worn out and expires."

The cause of this disease, Churchill attributes to cold, and an epidemic influence; yet he says, "I have seen a family of these little girls simultaneously attacked without any special, general, or local cause." I am not aware of its having prevailed as an epidemic in the United States. I had two cases during the past summer, both of which readily healed by external applications.

I was consulted August 15, 1867, by Mrs. R—— about her daughter, four years of age, on account of painful and spasmodic efforts in passing her urine. I prescribed for her, but getting no better, I was sent for next day, and found her in the following condition: Pulse 100; the labia swollen and inflamed externally, and excoriated internally; the orifice of the vagina inflamed and discharging a thin, whitish fluid. She was restless and crying from the pain and irritation. I ordered the bowels to be moved with oil: gave tincture ferri chloridi, and ordered external applications to the inflamed parts. From the 17th to the 23d of August the local disease remained stationery, and seemed to defy all external applications. Opiate lotions, poultices, alum, borax, tannin, liquor plumbi subacetas dilutus, all were in turn tried without giving any relief. The patient had no comfort except when under the influence of Dover's powder. As I entered her room on the 24th, I saw unmistakable evidence of an erysipelatous virus diffusing itself, by the aid of the aplastic condition of her system. The lips had a purplish hue, tongue brown and dry, pulse 130. The whole of the vulva was in an ulcerated condition. Aphthæ had spread around the anus, and over the perineum. The child having a hoarse cry, my attention was called to the fauces. I found them covered with aphthæ, and the tonsils covered with a diphtheritic exudation. The appetite was entirely lost. I put her upon the following treatment:

℞ Syr. Zingib. ℥ ij.

Aqua pura ℥ i.

Tr. Ferri Chlor. ʒ ij.

Potassæ Chloratis ʒ ii.

*M.*—A teaspoonful every 4 hours, alternated by a teaspoonful of the following:

℞ Aqua Cinnamomi ℥ i.

Sodæ Sulphitis ℥ ss.

Syr. Simp. ℥ ii.

*M.*—This was given through the day, and one grain of quinine and one of Dover's powder given every 4 hours during the night, unless she was asleep. The diet consisted of beef tea and milk porridge. I touched the ulcerated surfaces with a solution of nitrate of silver, and ordered a poultice of chamomile flowers to be applied to the parts.

25th.—In same condition. I touched the ulcers with dilute nitric acid, and continued the other treatment.

26, 27, 28th.—The chlorate potassa has increased the oxygen of the system, and thereby restored a healthy color to the lips and face. The iron and sulphite of soda have increased the plasticity of the blood; known by the drying and scaling-off of the vesicles about the lips. The ulcers of the vulva and perineum remain painful. I ordered the parts to be bathed, three times per day, and black-wash applied after each bathing.

29, 30th.—The ulcers look a little more healthy after using the black-wash, but are still painful. I had the parts thor, oughly cleansed with tepid water and milk, and after drying-covered the inflamed parts with the following,

℞—Collodion ℥ ss.

Glycerine gtt x.

Iodine gr. v.—*M.*

The application gave considerable pain for half an hour, after which she fell asleep, and slept for several hours; the first quiet and refreshing sleep she had had since first taken sick. The glycerine renders the collodion more supple, and less apt to crack when the parts are moved. The iodine acted as a disinfectant to the exhalations from the parts, and in a measure destroyed the septic poison, and neutralized the alkalies of the fluids of the parts which were being reabsorbed; and bringing the blood into that aplastic condition in which the tissues are rendered so sensitive to, and readily dissolve at the approach of the erysipellatous virus. I continued the tincture of iron, and increased the dose of the sulphite.

31st.—The ulcers looked clean and healthy. She rested better last night.; swelling and inflammation of the labia abating; pulse 100. Continue treatment.

Sept. 1st.—Quite comfortable; labia look healthy and natural; ulcers on the perineum dry and healing; skin moist.

3d.—Had some fever during the afternoon of the 2d, and very restless during the night, and complained of pain in the lower part of the abdomen. The orifice of the vagina looks highly inflammed and discharging a thin, whitish fluid. She is very much prostrated; pulse 130. Gave beef tea, alternated with the chlorate potassa and again applied the black-wash.

4th.—Very restless during the night, with pain in the stomach and bowels; vomited frequently; pulse weak and quick; extremeties cold. Gave wine whey, alternated with beef tea. I visited her again at 4 p. m., and found that the wine and beef essence had aroused the circulation, and the pulse was full and more regular; the extremities were warm.

5th.—Pulse imperceptible; extremities cold. She expired at 10 a. m. The patient had always been healthy, and gave evidence of the plastic diathesis up to the time her parents removed to 12th Street, between Market and Jefferson, in the spring of 1867. The gutters on that street have been in a filthy condition all summer; the waste-water from the houses above standing in seething, stagnant pools. This child played, all summer, under the shade of the trees covering that laboratory of zymotics.

We have in these cases a very interesting subject for investigation. Are they caused by the same epidemic influence, acting on an aplastic constitution; or does the epidemic carry with it a different degree of septic poison, which produces a difference in the degree of its malignancy?

I believe that the question is settled, that an epidemic disease manifests its degree of malignancy in proportion to the aplasticity of the blood, and not in the malignancy of the virus. For variola may be distinct in one neighborhood, and carried into another and prevail as confluent or malignant, owing to the sanitary condition of the place, a peculiar state of the atmosphere, reducing the inhabitants to an aplastic condition.

In all malignant epidemics, there are two morbid elements present, which give to the disease its degree of malignancy. One is the aplastic diathesis, and the other is the erysipelatous virus. The latter cannot exist in the system without the former; that is to say, the erysipelatous virus seldom or never attacks the plastic constitution; for immediately after its reception into the system, by inoculation or otherwise, the virus is surrounded by a wall of solid, plastic lymph, and carried out of the system, or made to localize itself, and the poison is expelled from the part by an abcess. But if the erysipelatous virus accompany the epidemic, or is produced by a chemical change of the animal tissues, and attack an aplastic constitution, it is very destructive to the tissues. They melt before its touch, like snow before the summer's sun. But virulent as they are, if the physician is on his guard, and watching their approach, they may be shorn of much of their terrors; for he has, in the tinctura ferri chloridi and the sulphites, together with sanitary regulations, almost a specific for aplastic and zimotic diseases. All physicians who have had much experience in the treatment of erysipelas by the tincture of iron have ceased to feel alarmed when that disease is prevailing. The surgeon no longer dreads erysipelas, phlebites, and pyæmia after an operation; for he has a prophylactic in the tincture of iron. Many of our best surgeons now precede their operations by the use of the iron, especially if the constitution is aplastic, and continue its use until the wound is healed. When this precaution is observed, it is seldom that either of thsse complications arises. The tincture of iron is not only valuable in restoring plasticity to the blood, but it destroys the catalytic agent by which zymotic diseases are generated and conveyed through the system. Yet valuable as it is in this respect, it is probably inferior to the sulphites of lime and soda. Paoli has demonstrated the fact that the sulphites, or the bi-sulphites, do check zymosis, and thereby retard or destroy the progress of that class of diseases depending upon a virus for their propagation.

As vulvites is most likely to attack an aplastic constitution, and to produce disintegration and ulceration of the tissues, thereby causing a chemical change of the fluids out of which the erysipelatous virus is frequently generated, we may expect more from the early use of the tincture of iron and the sulphites, than from all other remedies combined.

