# Omission

**Published:** 1867-10

**Authors:** 


### OMISSION.

The following paragraph was omitted in the making up of the first form. It is the last paragraph of the article by Dr. A. Given, of Louisville, Ky.:

They should be given in as large doses as the stomach will tolerate, and repeated every two hours. The inflamed and ulcerated parts should be cleansed with tepid water, thoroughly dried, and covered with the collodion glycerine, and iodine twice a day. Every practitioner is familiar with the rapid resolution of erysipellatous inflammation after the application of tincture of iodine and glycerine. Bromine possesses similar properties. But it must be borne in mind that external applications amount to but little, unless the aplasticity of the blood is corrected.

